# Supplementary material for: Mat thickness associated with Didymosphenia geminata and Cymbella spp. in the southern rivers of Chile
Source: PeerJ. 2019 Feb 15;7:e6481. doi: 10.7717/peerj.6481 (PMC6381927; doi:10.7717/peerj.6481)
Supplement: Supplemental Information 1 — Figure S1 and Table S1. [file peerj-07-6481-s001.docx]

Figure S1. Microscopic and macroscopic photographs of mucilage and mat present in Biobío river. A) Mucilage tubes generated by *Cymbella* spp. B) Mucilage stalk generated by *D. geminata.* C) Mucilage stalk produced by *Gomphoneis* spp. This genus did not have a relationship with mat thickness (unpublished data) (D) Mat in boulders. Scales of 10 μm.
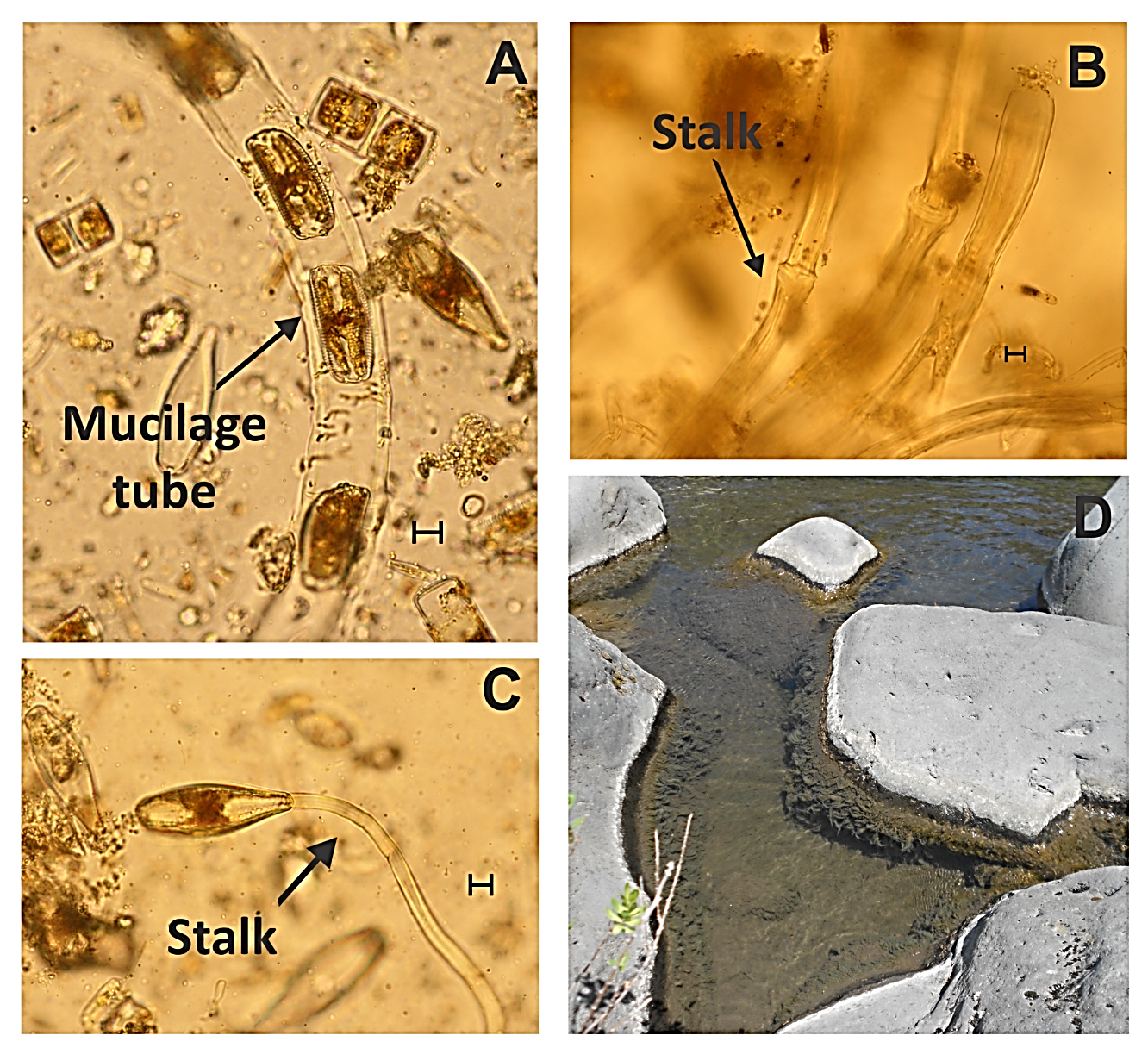


Table S1. Tables of ANOVAS and MANOVAS with permutations.

| Permutation ANOVA 1 way | | | | | | |
| --- | --- | --- | --- | --- | --- | --- |
| Resp. Var.: Mat thickness | Df | Sum Sq | Mean Sq | F value | P param | P perm |
| Categories *D. geminata/Cymbella* spp. Presence (A/A – C/A – A/D – C/D) | 3 | 2.73 | 0.91 | 3.39 | 0.02 | 0.02 |
| Residuals | 119 | 31.93 | 0.27 |  |  |  |
| Permutation MANOVA 1 way | | | | | | |
| Resp. Var.: Physico-chamical matrix | Df | Sum Sq | Mean Sq | F value | P perm |  |
| Sampling season (Spring - Autumn) | 0.25098 | 0.25 | 1 | 16.66 | 0.001 |  |
| Residuals | 1.59654 | 0.02 | 106 |  |  |  |
| Permutation MANOVA 1 way | | | | | | |
| Resp. Var.: Physico-chamical matrix | Df | Sum Sq | Mean Sq | F value | P perm |  |
| Categories *D. geminata/Cymbella* spp. Presence (A/A – C/A – A/D – C/D) | 0.13671 | 0.05 | 3 | 2.80 | 0.001 |  |
| Residuals | 1.69444 | 0.02 | 104 |  |  |  |
| Permutation ANOVA 1 way | | | | | | |
| Resp. Var.: SiO2 | Df | Sum Sq | Mean Sq | F value | P param | P perm |
| Categories *D. geminata/Cymbella* spp. Presence (A/A – C/A – A/D – C/D) | 3 | 22.08 | 7.36 | 9.01 | 0.00 | 0.001 |
| Residuals | 104 | 84.92 | 0.82 |  |  |  |
| Permutation ANOVA 1 way | | | | | | |
| Resp. Var.: Temp. Water | Df | Sum Sq | Mean Sq | F value | P param | P perm |
| Categories *D. geminata/Cymbella* spp. Presence (A/A – C/A – A/D – C/D) | 3 | 17.10 | 5.70 | 6.59 | 0.00 | 0.001 |
| Residuals | 104 | 89.90 | 0.86 |  |  |  |
| Permutation ANOVA 1 way | | | | | | |
| Resp. Var.: P | Df | Sum Sq | Mean Sq | F value | P param | P perm |
| Categories *D. geminata/Cymbella* spp. Presence (A/A – C/A – A/D – C/D) | 3 | 16.50 | 5.50 | 6.32 | 0.00 | 0.002 |
| Residuals | 104 | 90.50 | 0.87 |  |  |  |
| Permutation ANOVA 1 way | | | | | | |
| Resp. Var.: EC | Df | Sum Sq | Mean Sq | F value | P param | P perm |
| Categories *D. geminata/Cymbella* spp. Presence (A/A – C/A – A/D – C/D) | 3 | 15.13 | 5.04 | 5.71 | 0.00 | 0.002 |
| Residuals | 104 | 91.87 | 0.88 |  |  |  |
| Permutation ANOVA 1 way | | | | | | |
| Resp. Var.: DO | Df | Sum Sq | Mean Sq | F value | P param | P perm |
| Categories *D. geminata/Cymbella* spp. Presence (A/A – C/A – A/D – C/D) | 3 | 5.48 | 1.83 | 1.87 | 0.14 | 0.123 |
| Residuals | 104 | 101.52 | 0.98 |  |  |  |
| Permutation ANOVA 1 way | | | | | | |
| Resp. Var.: Fe | Df | Sum Sq | Mean Sq | F value | P param | P perm |
| Categories *D. geminata/Cymbella* spp. Presence (A/A – C/A – A/D – C/D) | 3 | 3.65 | 1.22 | 1.22 | 0.30 | 0.336 |
| Residuals | 104 | 103.35 | 0.99 |  |  |  |
| Permutation ANOVA 1 way | | | | | | |
| Resp. Var.: pH | Df | Sum Sq | Mean Sq | F value | P param | P perm |
| Categories *D. geminata/Cymbella* spp. Presence (A/A – C/A – A/D – C/D) | 3 | 1.59 | 0.53 | 0.52 | 0.67 | 0.684 |
| Residuals | 104 | 105.41 | 1.01 |  |  |  |
| Permutation ANOVA 1 way | | | | | | |
| Resp. Var.: Depth flow vel. | Df | Sum Sq | Mean Sq | F value | P param | P perm |
| Categories *D. geminata/Cymbella* spp. Presence (A/A – C/A – A/D – C/D) | 3 | 0.26 | 0.09 | 0.08 | 0.97 | 0.971 |
| Residuals | 104 | 106.74 | 1.03 |  |  |  |
| Permutation ANOVA 1 way | | | | | | |
| Resp. Var.: Ca | Df | Sum Sq | Mean Sq | F value | P param | P perm |
| Categories *D. geminata/Cymbella* spp. Presence (A/A – C/A – A/D – C/D) | 3 | 3.96 | 1.32 | 1.33 | 0.27 | 0.273 |
| Residuals | 104 | 103.04 | 0.99 |  |  |  |
| Permutation ANOVA 1 way | | | | | | |
| Resp. Var.: Water column depth | Df | Sum Sq | Mean Sq | F value | P param | P perm |
| Categories *D. geminata/Cymbella* spp. Presence (A/A – C/A – A/D – C/D) | 3 | 3.44 | 1.15 | 1.15 | 0.33 | 0.329 |
| Residuals | 104 | 103.56 | 1.00 |  |  |  |
